# Supplementary material for: Metabolome-Based Classification of Snake Venoms by Bioinformatic Tools
Source: Toxins (Basel). 2023 Feb 15;15(2):161. doi: 10.3390/toxins15020161 (PMC9963137; doi:10.3390/toxins15020161)
Supplement: Supplementary file 1 [file toxins-15-00161-s001.zip › Documents S.1.pdf]

Supporting Information – Documents S.1

Section 1 – Further information regarding Principal Component Analysis:

To perform PCA, the first step would be to calculate the covariance matrix of the dataset. This matrix is a square matrix that holds in each of its positions the covariance between each pair of vectors within the matrix -thus, the diagonal of this matrix will contain the variances of said vectors. Once the covariance matrix has been obtained, the eigenvalues of said matrix can be extracted and sorted out from the highest to the lowest value (as they represent how much of the overall variance they explain). At the same time, a matrix whose columns are the corresponding eigenvectors of said eigenvalues is extracted. With this procedure we can obtain orthonormal vectors, each of which holds information of all the variables. The information that they contain is the weight that each of the variables has in said eigenvector. Thus, we can obtain a matrix holding in its columns the weight that each of the variables has in each eigenvector, with said columns ordered by how much variance of the overall model is each column able to explain. These columns are the so-called Principal Components (PCs) of the data, and the number of PCs that this matrix has can be chosen and ordered based on how much variability is explained only with said columns. The matrix containing these PCs is defined as the *Loadings* (**P**) of the PCA model.

Once **P** has been defined, the data set (called **X** from now on) can be projected onto **P,** rendering another matrix defined as *Scores* (**T**). Thus, *Equation 1* can be defined:

$$\boldsymbol{T} = \boldsymbol{XP} \qquad \textit{Eq. 1}$$

And *Equation 2* should be satisfied:

$$\boldsymbol{X} = \boldsymbol{TP^T} + \boldsymbol{E} \qquad \textit{Eq. 2}$$

For P^T being the transposed matrix of P and E being the matrix resulting from the part of the data that is not explained by **P**. The part of **X** that **P** and **T** can explain will be referred to as the model ($\widehat{\boldsymbol{X}}$) and must satisfy *Equation 3:*

$$\widehat{\boldsymbol{X}} = \boldsymbol{TP^T} = \boldsymbol{X} - \boldsymbol{E} \qquad \textit{Eq. 3}$$

Thus, **X** is the original dataset, **P** is a matrix that contains information about each of the variables in each of its columns, **T** is a matrix that holds information about how **X** interacts with **P**, and **E** is a matrix that contains information about the data that the model is not able to explain.

Section 2 - Further information regarding k-means clustering modelling:

k-clustering consists of classifying a given *n* number of samples by analyzing their *Scores* and grouping them with the cluster that contains the mean that is the closest to said *Scores*. The measured distances were the Euclidean ones, thus *Equations 4* and 5 were applied.

$$Distance\ to\ Viperidae\ cluster's\ mean\ =\ d_V\ =\sqrt{\sum_{1}^{n}\left((cluster's\ mean_{Viperidae} - scores_n)^2\right)} \quad \text{Eq. 4}$$

$$Distance\ to\ Elapidae\ cluster's\ mean\ =\ d_E\ =\sqrt{\sum_{1}^{n}\left((cluster's\ mean_{Elapidae} - scores_n)^2\right)} \quad \text{Eq. 5}$$

If $d_E < d_V$ the sample is classified as *Elapidae.* If $d_V < d_E$ the sample is classified as *Viperidae.*

The *Bootstrapping* step revealed that the appropriate value for the *Rep* variable was 36, as in 72 of the 100 iterations all the extracted values -used as samples- were correctly classified. This was the highest number of correctly-assigned bootstraps. This leads to the model being created with 7 variables.

The *V1* step revealed that the number of PCs that could be used to correctly assign the first test set was every number in between 3 and 7. However, if $\boldsymbol{X} = \boldsymbol{\hat{X}}$ **,** there is a high chance of overfitting. Thus, the variance explained by the model can be plotted against the number of PCs to choose the number of PCs that will explain most of the model of the variance without creating a model that will overfit the data. This is called "Scree plot" and it can be found in *Fig A.1.*

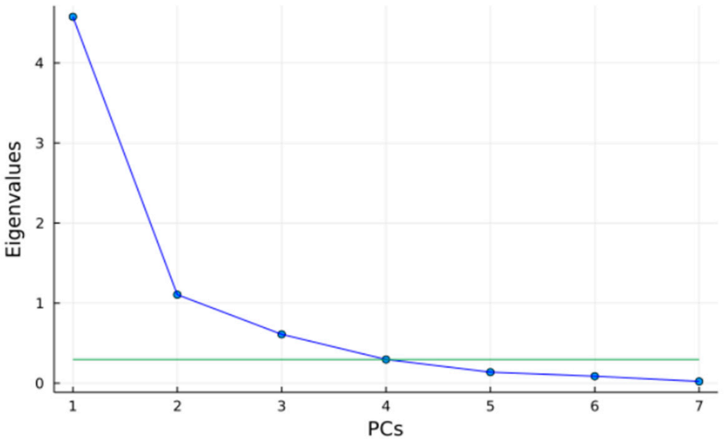

Figure A.1.- Scree plot of the PCA for Rep = 36. The elbow is marked with a green line. This graph shows the number of PCs that should be considered to explain most of the variability within the model without overfitting the data.

The chosen number of PCs was 4 because the mentioned elbow can be seen at that point of the Scree plot, meaning that the explained variance from that point on becomes much smaller –thus, much less relevant.

Section 3 – 1st Validation of the k-means clustering model:

The *V1* step revealed that the number of PCs that could be used to correctly assign the first test set was every number in between 3 and 20. However, if $\boldsymbol{X} = \widehat{\boldsymbol{X}}$, there is a high chance of overfitting. Thus, the eigenvalues can be plotted against their corresponding PC to choose the number of PCs that will explain most of the model of the variance without creating a model that will overfit the data. This type of plot is defined Scree plot, and the chosen number of PCs is chosen based on the "elbow" that appears, which represents the point where the addition of more PCs does not add much to the explanation of the overall model. This can be found in *Fig A.2.*

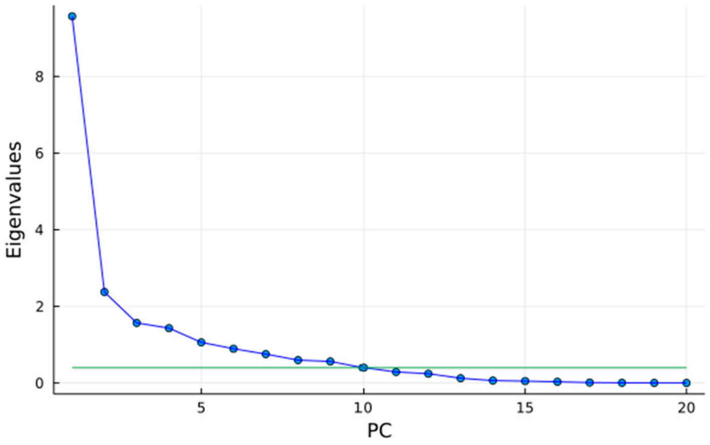

Figure A.2.- Scree plot of the PCA for Rep = 20 The elbow is marked with a green line. This graph shows the number of PCs that should be considered to explain most of the variability within the model without overfitting the data.

The chosen number of PCs was 10 because the mentioned elbow can be seen at that point of the Scree plot, meaning that the explained variance from that point on becomes much smaller –thus, much less relevant.

Section 4 – Results of the k-means clustering model:

As it has been mentioned, a classifier based on *k*-means clustering with *Rep* = 36 and nº PCs = 4 was built. A 2D representation of this model including the *Scores* projected onto the two first PCs can be found in *Figure 2*. The cluster's centers have also been included in said image. 2 PCs have been chosen for this representation as it is easier to visualize, but the actual subspace is contained in 4 dimensions, as they were the ones chosen to describe most of the variance of the model.

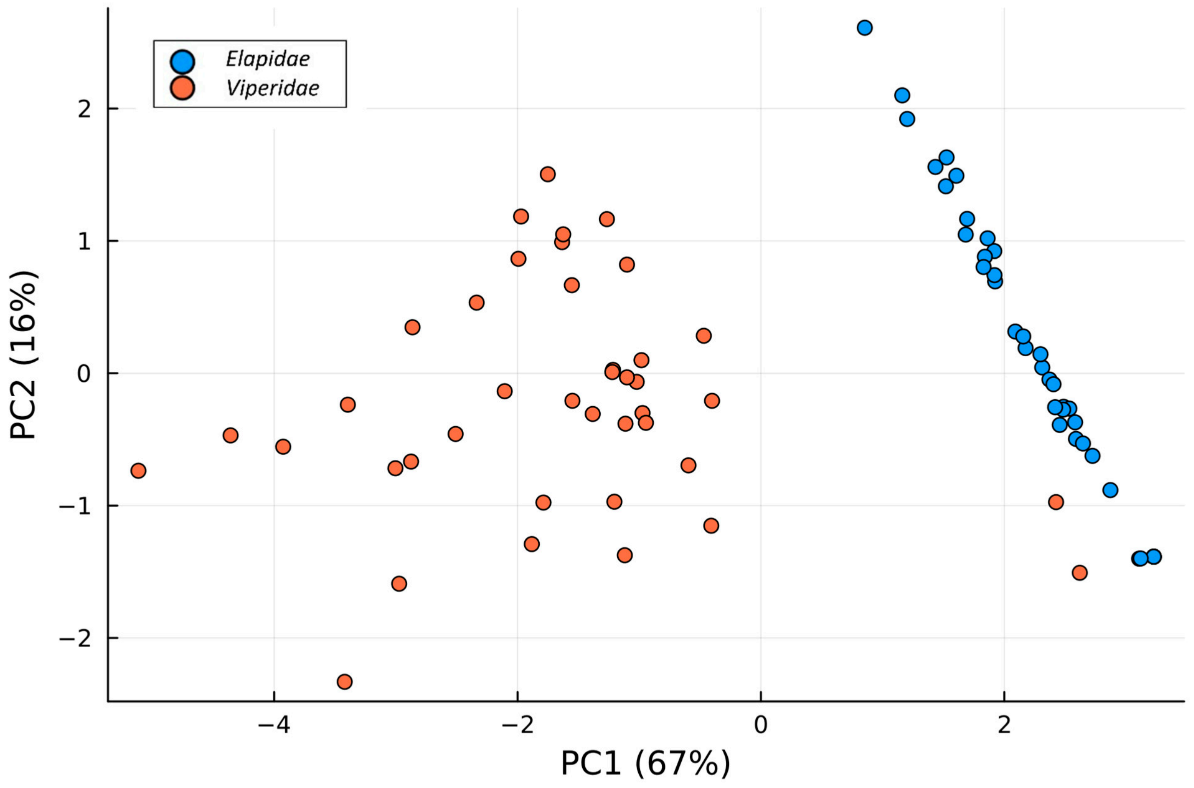

Figure A.3.- 2D representation of the scores coming from the k-means model for Rep = 36 and nº PCs = 4 of PCs 1 and 2. Red colors come from Viperidae snakes while blue and white colors come from Elapidae ones.

At first glance it would seem like both families separate nicely, although the scattering of the *Viperidae* could end up being a problem due to the amorphous distribution of its cluster. It would be interesting to also mention how the non-spitting *Najas* seem to cluster around the bottom of the *Elapidae* distribution.

As it can be seen, the 1st PC is the one that better separates both families -as the projection of the scores onto this axis would allow for classification of most of the samples. However, by looking at its loadings in *Figure A.4.*, it seems like all features affect in a similar way to said component. In said figure we can find the values of the value of the *Loadings* for each PC and variable, divided by the mentioned sum, and multiplied by 100 to obtain, in a percentage, the extent to which feature affects each PC.

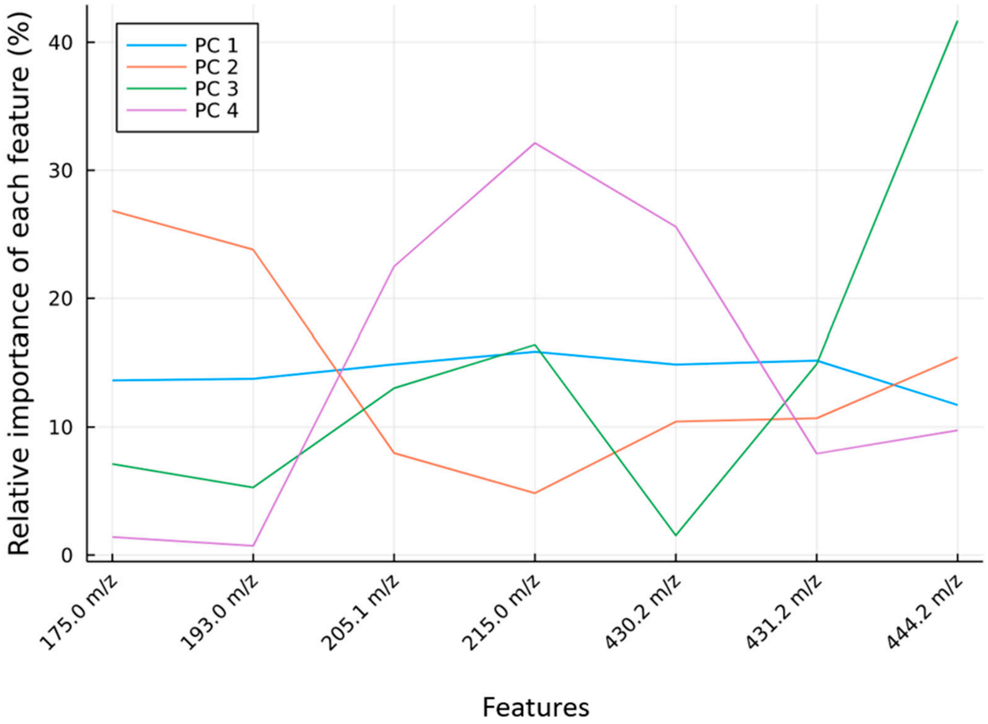

Figure A.4.- Relative importance of each feature in the PCs obtained from the model for Rep = 36. The higher the percentage, the more repercussion the variables have in the PCs. The variance explained by every PC becomes smaller the further the PC we investigate.

The most important variables in the PCs are those that generate the most intense change in the variance of the PC -or, visually, the values that differ the most from 0. As it can be seen, all of the PCs are strongly affected by several features. This means that there is not a "master feature" able to differentiate between *Elapidae* and *Viperidae* snakes, but rather all of them help in this goal. The 1st PC is the one that better separates both families -as the projection of the scores onto this axis would allow for classification of most of the samples. However, it seems like all features affect in a similar way to said component. When looking at the 2nd PC, it can be seen that it is mostly defined by the first two features (175.0 & 193.0 *m/z*). This component -as shown in *Figure 6*-, is the one that defines most of the variability seen within the *Elapidae* family, and the same could be said for the *Viperidae* although to a lesser extent.

When looking at the 2nd PC, it can be seen that it is mostly defined by the first two features (175.0 & 193.0 *m/z*). This component -as shown in *Figure 2*-, is the one that defines most of the variability seen within the *Elapidae* family, and the same could be said for the *Viperidae* although to a lesser extent. The autoscaled values of those 7 features has been summarized in a heatmap that is depicted in *Figure 3*.

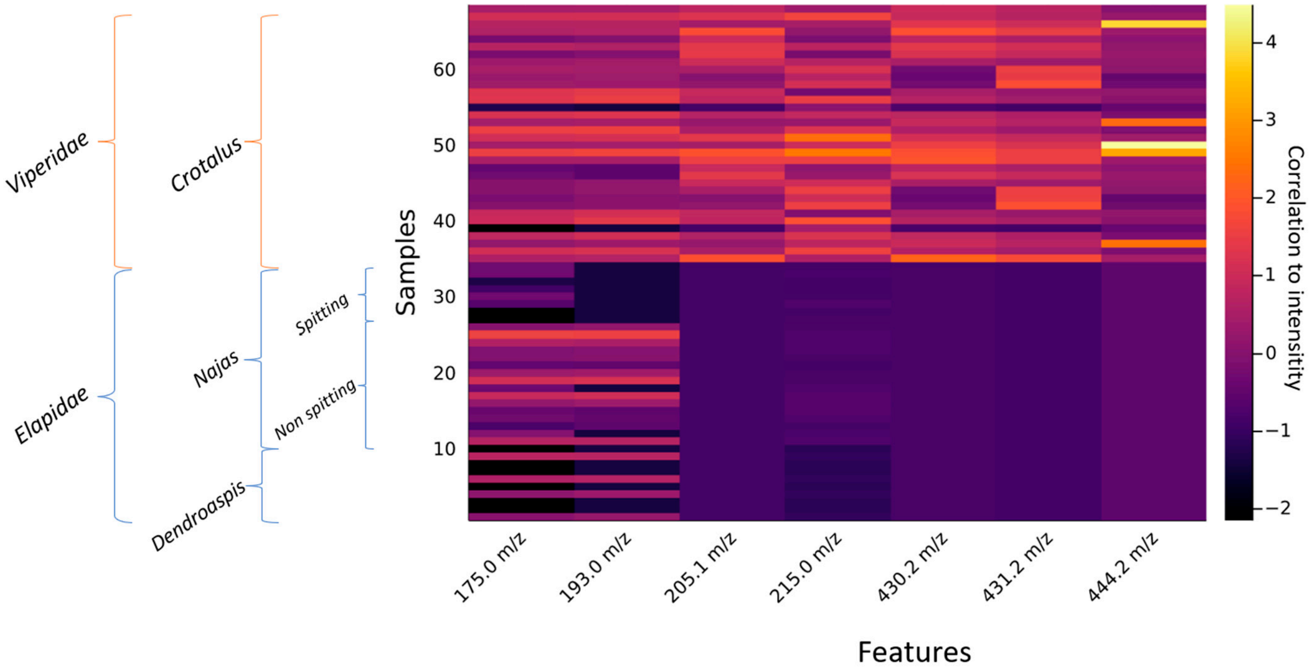

Figure A.5.- Heatmap of the autoscaled matrix for the PCA with Rep = 36. 7 Features are considered relevant and, in the graph, a correlation between the color and the autoscaled data can be seen (lighter colors mean more signal and darker colors mean less signal). The subspecies have also been classified for biological relevance based on their family and genus.

Taking into account that the first 34 samples correspond to *Elapidae* snakes and the other half to *Viperidae,* it can be inferred why do we see such clustering in *Figure A.3*. While *Viperidae* seems to hold considerable amounts of the features 3-7 (from 200 to 450 *m/z*) the same cannot be said for *Elapidae,* which is mainly defined by only the first two features (from 170 to 200 *m/z*). This is the reason as to why most of the variance seen within the *Elapidae* family is found in the 2nd Principal Component, as it's the only one that gives importance to those two features. Within the *Elapidae,* the *Dendroaspis* genus (which has positive values for PCs) seems to be quite similar to the *Naja* genus, only differing in the intensity of the 1st feature (175.0 *m/z)*, which is not as common in *Dendroaspis* as in *Najas,* and the 2nd feature (193.0 *m/z*), which is much more common in *Dendroaspis* compared to *spitting Najas*, but much less common in *Dendroaspis* compared to *non-spitting Najas.* Because the *non-spitting Najas* don't hold much intensity for neither of the first two features, they cluster around a value of 0 for the 2nd PC.

In the *Figure A.6* the scores for the test-set have also been included. Because of time-related issues and the amount of available venom, all the samples in the test-set come from *Viperidae* snakes.

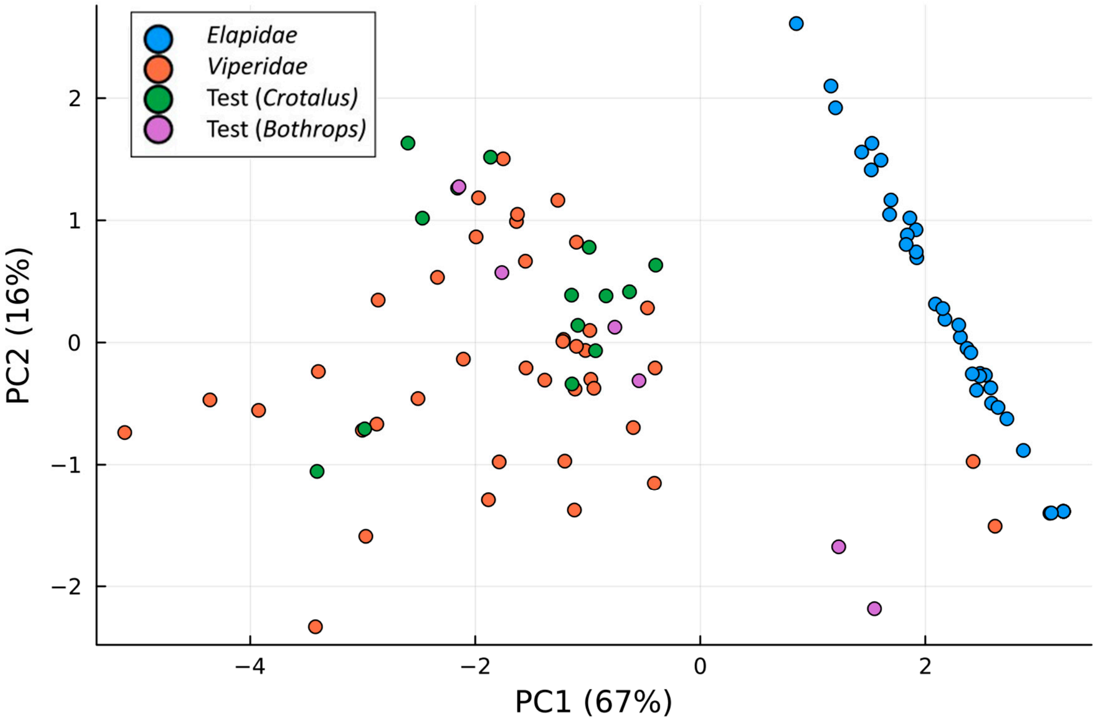

Figure A.6.- 2D representation of the scores coming from the model and from the validation set for Rep = 36 and nº PCs = 4 of PCs 1 and 2. Red colors come from Viperidae snakes while blue and white colors come from Elapidae ones.

The next graph -*Figure A.7.*- shows how the model classified the introduced samples by means of the classification explained in *Table 2* in the A.I.

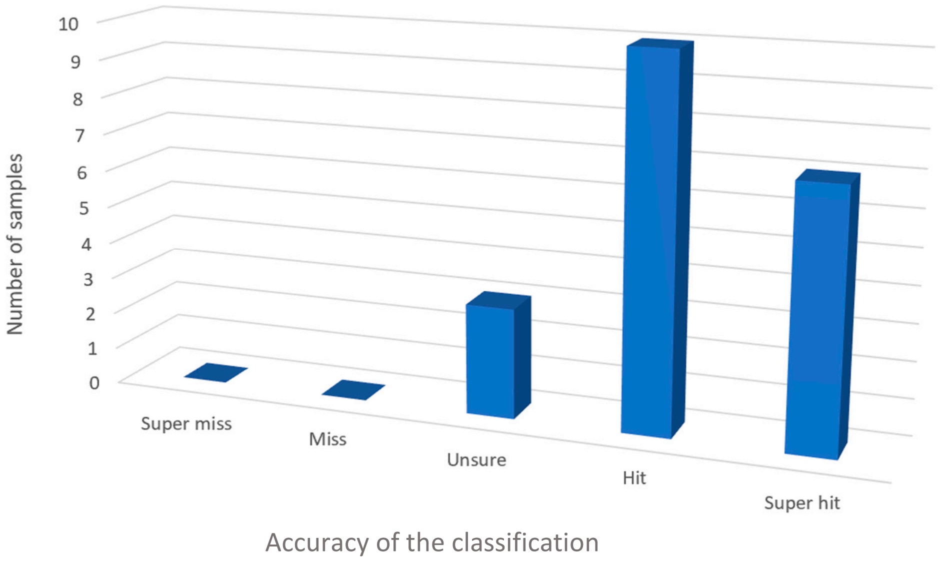

Figure A.7.- Graphical representation of the classification ability of the created model. It did not classify incorrectly any of the species, but it was also not completely sure about how to classify most of the samples.

Section 5 – Representation and analysis of the Loadings of the SVM model:

To understand the information held in the PCs, their *Loadings* are represented. To do this, the absolute values of the *Loadings* of each PC were taken and summed. In *Figure A.8.* we can find the values of the value of the *Loadings* for each PC and variable, divided by the mentioned sum, and multiplied 100 to obtain, in a percentage, the extent to which feature affects each PC. Only the first principal components have been represented because they explain most of the variance (80%) of our system. Had the 10 PCs been taken into account, the graph would become too crowded, making it difficult to read.

Table A.3 of the *A.I.* contains the relative importance of each feature in all the PCs and the variance explained by each PC are included.

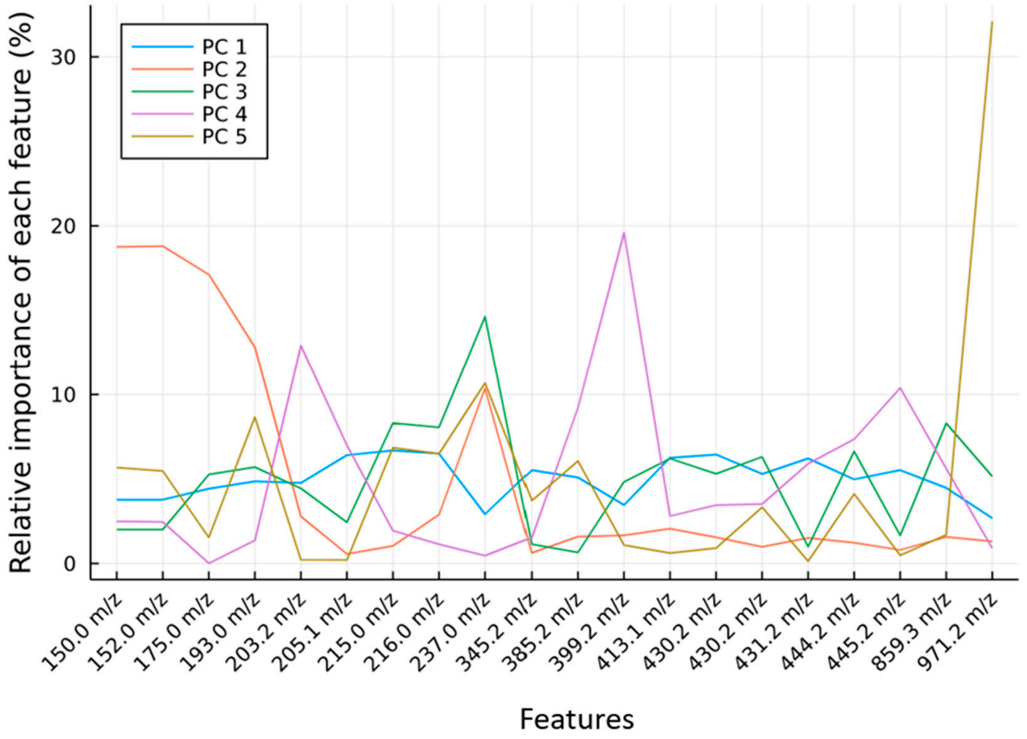

Figure A.8.- Relative importance of each feature in the PCs from the model for Rep = 20. The higher the percentage, the more repercussion the variables have in the PCs. The variance explained by every PC becomes smaller the further the PC we investigate.

The most important variables in the PCs are those that generate the most intense change in the variance of the PC -visually, the values that differ the most from 0. As seen for the model with *Rep* = 36, the first Principal Component gives a similar relevance to each of the features, using all of them to define most of the variance in the model. However, a deeper analysis of said PC will be presented in the next paragraph. As it also happened in the model for *Rep* = 36, the 2nd PC is mostly defined by the first set of features (4 in this case), but they differ in the last feature: while the latter mentioned model included feature 7 (445.1960 *m/z*) inside the 2nd PC, this model leaves one PC (the 5th one) to be mainly defined

by the last feature (971.1695 *m/z*). In general, it seems like, because this model has 10 PCs and not 4, it can distribute better the importance of each feature along the different components, allowing for a better explanation of the relevant model variation.

By looking more closely at the absolute values of the 1st PC for each variable, we could define what the classification is mostly based on. To get **T**, **X** is projected onto **P**, meaning that the higher the values of **P** we can find for each variable, the more important they are to classify *Elapidae,* whereas the lower they are, the more important they are to classify *Viperidae.* This is represented in *Figure A.*9.

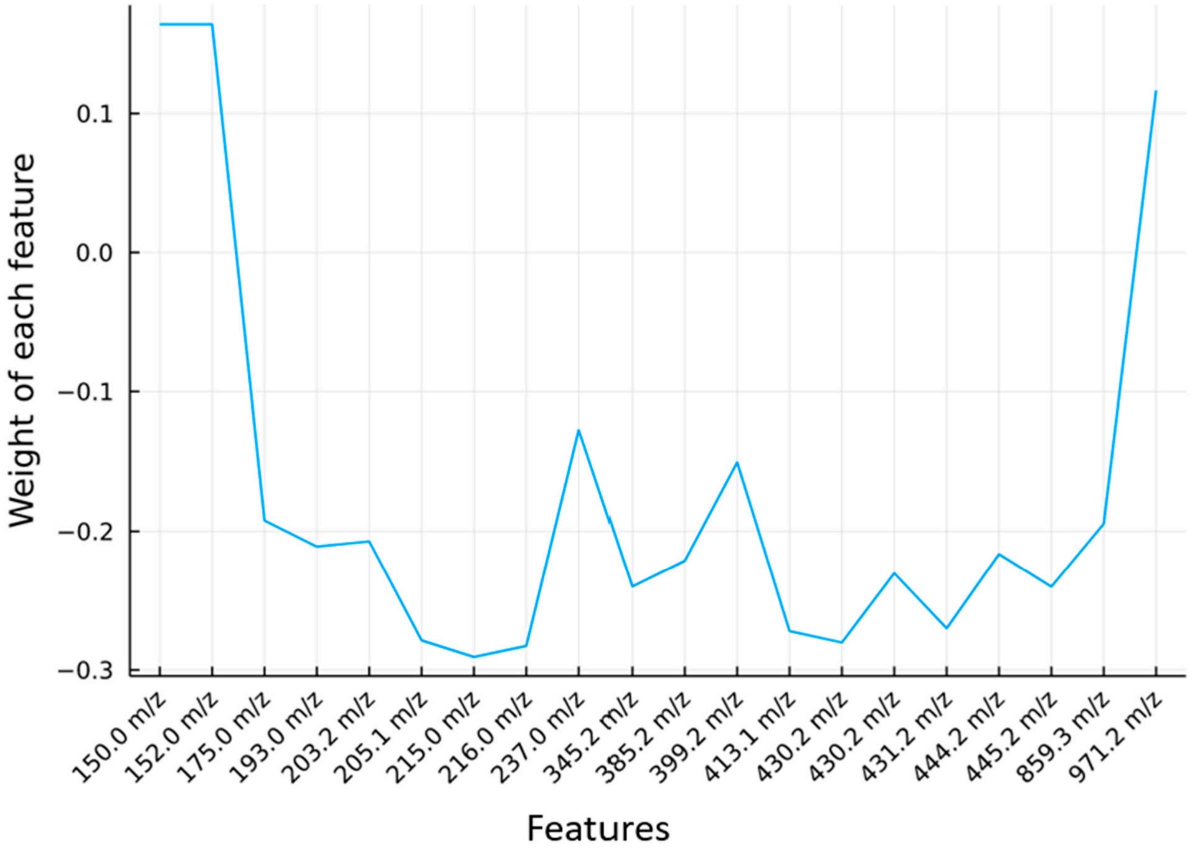

Figure A.9.- Weight of each of the variables for the 1st Principal Component for Rep = 20. The more the values differ from 0, the more important that variable it is for defining PC 1. If the weights are higher than 0, they are used for defining Elapidae. If the weights are smaller than 0, they are used for defining Viperidae

This graph indicates that, while features 1,2 and 20 (the ones with weights higher than 0) are only ones to be considered when classifying a sample as *Elapidae*, features 6, 7 and 8 (the ones with the lowest weights) are essential when classifying a sample as *Viperidae* and explaining most of its variance.

Section 6 - Thresholds applied on MSConvert:

Output format: mzXML

Binary enconding precision: 32 bits

Write index: Check

TPP compatibility: Check

Subset:

  Scan time(seconds): 720 - 2520

Threshold peak filter:

  Threshold type: Absolute intensity

  Orientation: Most intense

  Value: 500

Section 7 - Example of data visualization with the dashboard

The dashboard in Tableau allows for us to easily modify what we want to see and how we want to see it.

The representation of the data within the model would look like the example given in Figure A.10

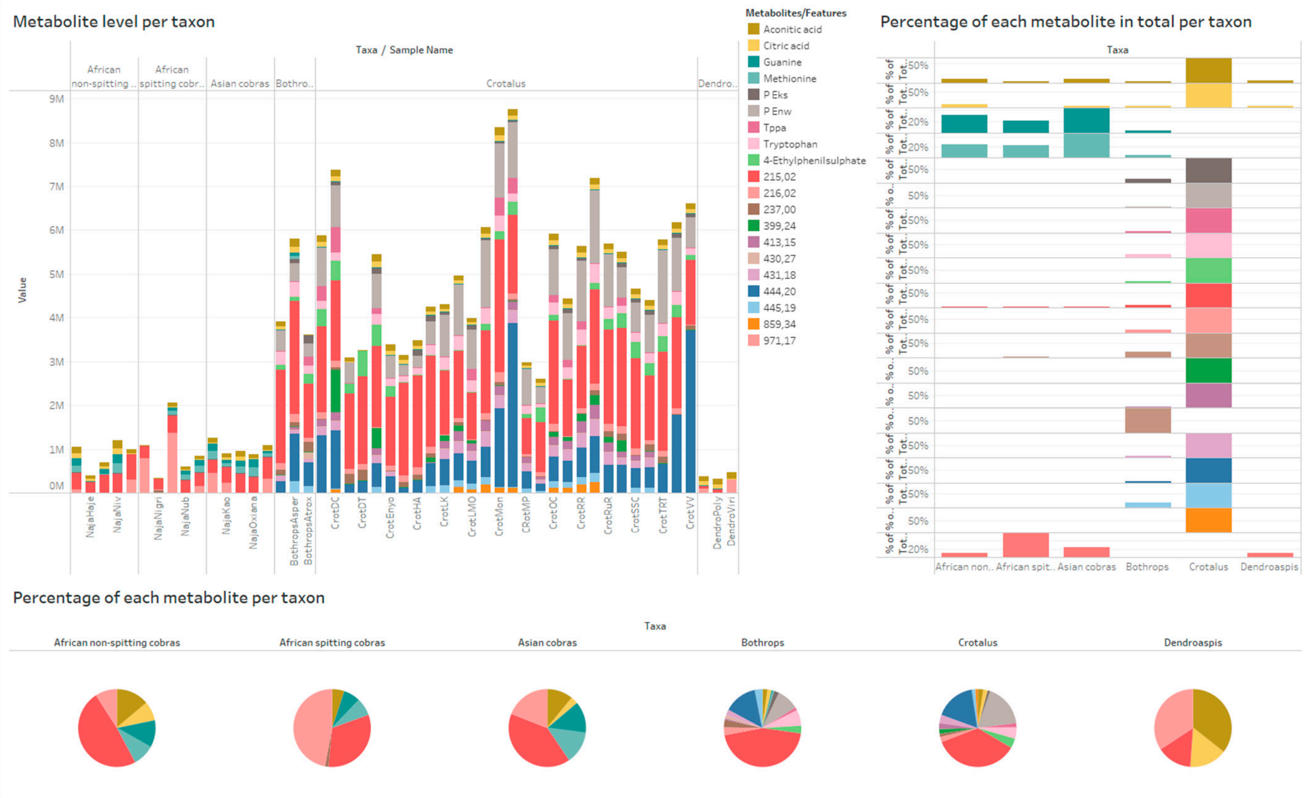

Figure A.10.- Tableau visualization of the dashboard containing information regarding all relevant metabolites for the model in the analyzed samples

But by clicking in the different metabolites we can highlight certain information, as can be seen in Figure A.11.

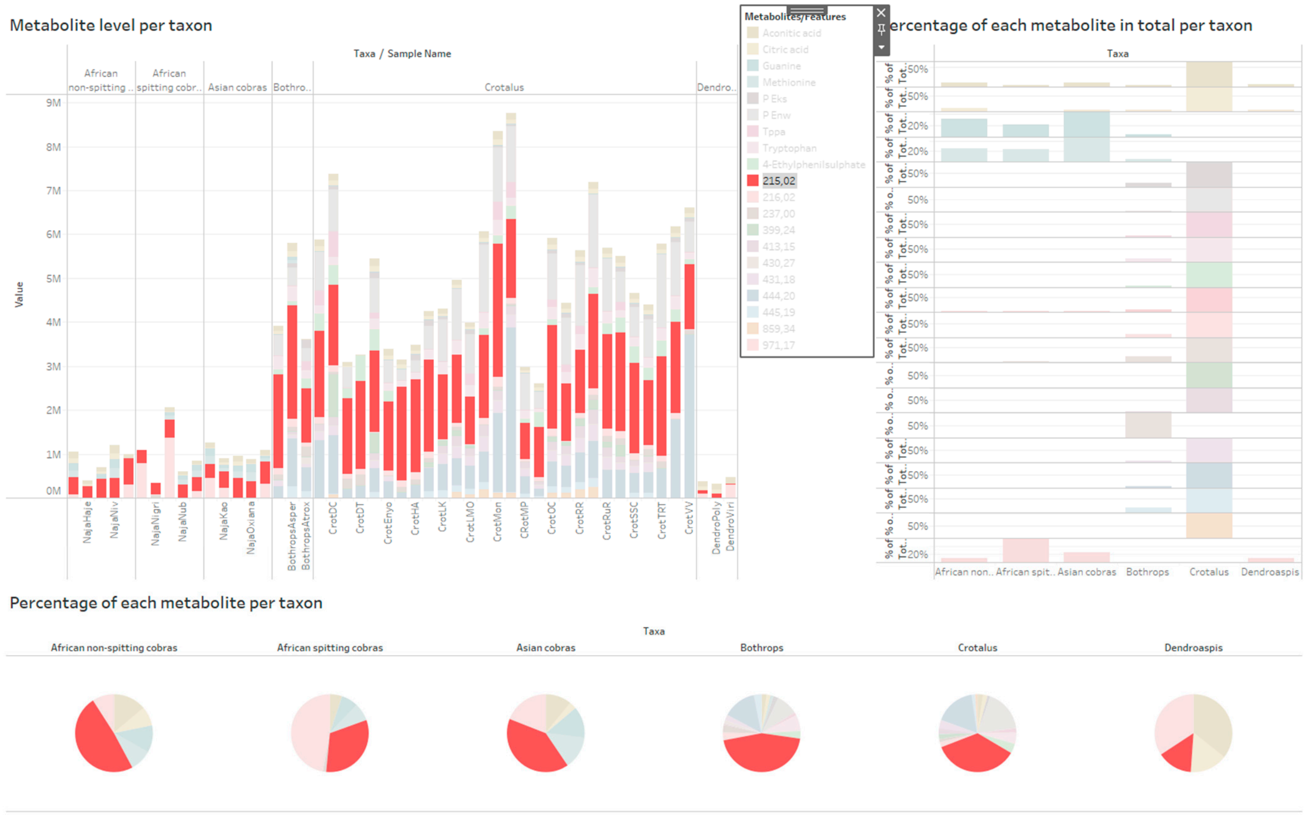

Figure A.11.- Tableau visualization of the dashboard containing information regarding all relevant metabolites for the model in the analyzed samples, when one of the metabolites is highlighted.

And we can look at each sample or taxon more in-depth by double clicking the sample or taxon we are interested in, as it can be seen in Figure A.12

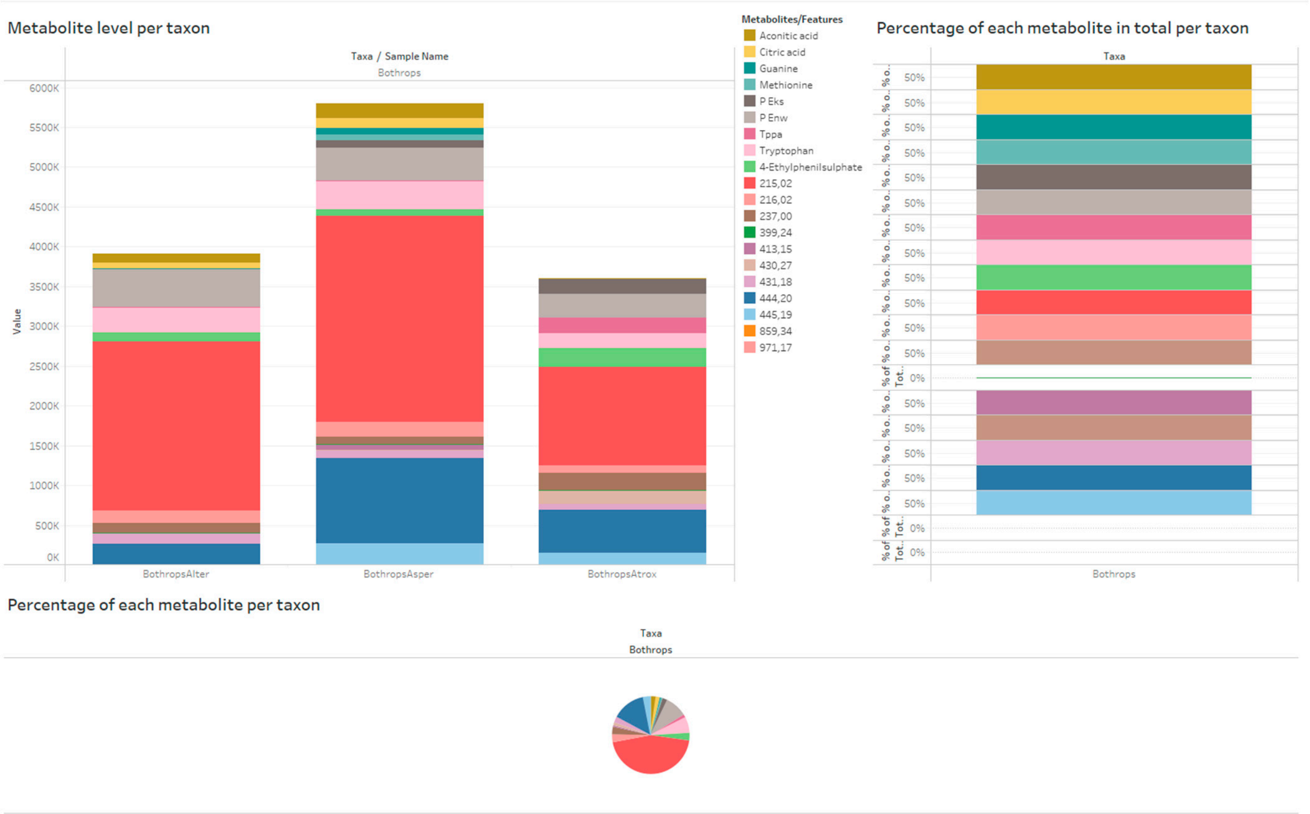

Figure A.12.- Tableau visualization of the dashboard containing information regarding all relevant metabolites for the model in the analyzed samples, when one of the taxa is highlighted.
